# Supplementary material for: Quitting Smoking before and after Pregnancy: Study Methods and Baseline Data from a Prospective Cohort Study
Source: Int J Environ Res Public Health. 2022 Aug 17;19(16):10170. doi: 10.3390/ijerph191610170 (PMC9408087; doi:10.3390/ijerph191610170)
Supplement: Supplementary file 1 [file ijerph-19-10170-s001.zip › ijerph-1728271-supplementary.pdf]

Table S1. Comparison among survey sample versus CDC 2018 Natality data.

Variable	Survey Sample (N = 62)	CDC (N=297.768)	Cohen's d Values
Age, n(%)			
<= 24	15 (24.2)	100412 (33.7)	0.26
25 – 29	12 (19.4)	99175 (33.3)	0.40
30 – 34	27 (43.6)	64744 (21.7)	0.56
>= 35	8 (12.9)	33437 (11.2)	0.09
Education, n(%)			
Less than high school	11 (17.7)	62689 (21.1)	0.12
High school/equivalent diploma	22 (35.5)	124634 (41.9)	0.15
Some college or tech school	21 (33.9)	94873 (31.9)	0.05
College graduate	7 (11.3)	12463 (4.2)	0.59
Master or doctor	1 (1.6)	3109 (1.0)	0.24
Hispanic/Latina, n (%)	3 (4.8)	21620 (7.3)	0.24
Race, n (%)			
Black or African American only	28 (45.2)	37413 (12.6)	0.96
White only	22 (35.5)	247345 (83.1)	1.21
Multiracial	12 (19.4)	13010 (4.4)	0.92
Married/partnered, n (%)	29 (46.7)	85126 (29.3)	0.16
WIC benefits receiver, n (%)	34 (54.8)	163972 (55.1)	0.01
BMI (kg/m^{2}Based on pre-pregnancy weight), n (%)			
Underweight/Normal (<= 24.9)	21 (33.9)	130724 (43.9)	0.23
Overweight (25.0 – 29.9)	13 (21.0)	72268 (24.3)	0.10
Obesity (>= 30.0)	28 (45.2)	94776 (31.8)	0.31
Cigarettes Per Day, mean (SD)	10.6 (7.1)	13.3 (10.4)	0.26
